# Using a very low-density SNP panel for genomic selection in a breeding program for sheep

**DOI:** 10.1186/s12711-017-0351-0

**Published:** 2017-10-24

**Authors:** Jérôme Raoul, Andrew A. Swan, Jean-Michel Elsen

**Affiliations:** 1 Institut de l’Elevage, Castanet-Tolosan, France; 2 GenPhySE, INRA, Castanet-Tolosan, France; 3 Animal Genetics and Breeding Unit, University of New England, Armidale, Australia

## Abstract

**Background:**

Building an efficient reference population for genomic selection is an issue when the recorded population is small and phenotypes are poorly informed, which is often the case in sheep breeding programs. Using stochastic simulation, we evaluated a genomic design based on a reference population with medium-density genotypes [around 45 K single nucleotide polymorphisms (SNPs)] of dams that were imputed from very low-density genotypes (≤ 1000 SNPs).

**Methods:**

A population under selection for a maternal trait was simulated using real genotypes. Genetic gains realized from classical selection and genomic selection designs were compared. Genomic selection scenarios that differed in reference population structure (whether or not dams were included in the reference) and genotype quality (medium-density or imputed to medium-density from very low-density) were evaluated.

**Results:**

The genomic design increased genetic gain by 26% when the reference population was based on sire medium-density genotypes and by 54% when the reference population included both sire and dam medium-density genotypes. When medium-density genotypes of male candidates and dams were replaced by imputed genotypes from very low-density SNP genotypes (1000 SNPs), the increase in gain was 22% for the sire reference population and 42% for the sire and dam reference population. The rate of increase in inbreeding was lower (from − 20 to − 34%) for the genomic design than for the classical design regardless of the genomic scenario.

**Conclusions:**

We show that very low-density genotypes of male candidates and dams combined with an imputation process result in a substantial increase in genetic gain for small sheep breeding programs.

## Background

Although technical and economic interests of genomic breeding programs in sheep have been positively assessed by various authors [1–5], only a few countries deliver genomic breeding values for meat and dairy sheep [6]. For many sheep breeds, the remaining main obstacle is to build an efficient reference population based on medium-density (MD ~ 50 K) genotypes and estimated breeding values (EBV) of sires to estimate the effects of single nucleotide polymorphisms (SNPs). Genomic prediction accuracy depends mainly on the number of animals included in the reference population, the accuracy of their EBV, their relationship with target animals (mostly candidates without phenotypes), and the effective size of the population [7]. In sheep, compared to dairy cattle, the effective population size is generally larger [8], and especially for maternal traits, EBV of both artificial insemination (AI) and naturally-mated sires are less accurate due to smaller numbers of progeny per sire. An increase in reference population size could counterbalance these unfavorable factors. However, this increase is limited when the population itself is small and the reference population is based on sires only. Including records and genotypes from lower tiers of the population is promising [9], whereas, so far, multi-breed approaches have not led to the expected increases in genomic prediction accuracy [10]. Another way to increase the reference population would be to include females. Studies on dairy cattle breeding programs show that this strategy is efficient, especially when the reference population is based on a limited number of sires and/or records [11–15]. The impact of including females in the reference population on a sheep breeding program has never been assessed.

In sheep, the profitability at the nucleus level remains a critical factor for the design of breeding programs [16]. Implementation of genomic selection is mostly likely only if the cost is similar to that of the current design. In a genomic design, AI sires are no longer progeny-tested but both the animals in the reference population and the selection candidates must be genotyped. Since genotyping costs are quite high in sheep relative to the economic value of breeding animals, the number of genotypes has a large influence on the profitability of the design. Including dams in the reference population would increase the genotyping costs. Besides, the magnitude of the selection differential on genomic estimated breeding values (GEBV) of male candidates is a critical factor that determines the additional benefit of genomic selection breeding programs [17]. Increasing the selection differential requires additional candidate genotypes resulting in additional genotyping costs as well.

Very low-density (VLD) genotypes in association with imputation techniques would reduce the genotyping costs of a genomic design. VLD panels based on a few hundred SNPs are available at lower cost than low-density genotypes (~ 15 K) and are mainly used for parentage assignment [18–20]. Since candidate SNPs for parentage assignment are widely available across breeds (e.g. 9269 SNPs had a minor allele frequency higher than 0.30 in at least 20 French sheep populations [20]), new panels under development will probably be close to 1000 SNPs. Imputation techniques based on common SNPs that are present on both MD and VLD panels can be used to infer missing MD genotypes of male candidates and dams, thus to exploit MD genotypes of selected sires as a reference population. Population-based methods use the linkage disequilibrium (LD) between SNPs and haplotype frequencies only, whereas population- and family-based methods also include co-segregation information based on pedigree. The factors that affect imputation accuracy [21–35] and the relation between imputation accuracy and genomic prediction quality [21, 25–27, 30, 31, 34, 35] are well documented. In sheep, Moghaddar et al. [31] found a correlation close to 1 between GEBV computed from real versus imputed genotypes with an average imputation accuracy of 0.96. The imputation accuracy depends on: (1) the characteristics of the low-density panel with respect to the minor allele frequency, the number, spacing and localization of SNPs [21–27, 30, 33, 34]; (2) the linkage between adjacent SNPs [22, 24]; and (3) the characteristics of the reference population including size, single or multi-breed population and relationship with imputed animals [21, 24, 26, 27, 29–31, 34]. As observed with genomic prediction accuracy, imputation accuracy increases as close relatives are included in the reference population and pedigree information is used [21, 24, 26, 29, 30, 34].

Focusing on a breeding program applied to a small population of purebred sheep in which both AI and natural mating sires are used, the objectives of this study were to quantify the impact of (1) increasing the reference population size with female genotypes and (2) imputing genotypes of male candidates and females from very low- to medium-density SNP panels. The presence of all sires and grand-sires of dams and male candidates in the reference population for imputation is expected to limit the detrimental effect of using a VLD panel on imputation accuracy. Various designs were compared, including a classical selection scheme based on progeny testing of AI sires as a baseline scenario, and several genomic selection schemes. Five scenarios were assessed for genomic selection by varying the reference population component (with or without dams), and genotype information (MD or imputed MD genotypes for male candidates and dams).

## Methods

**Fig. 1:**
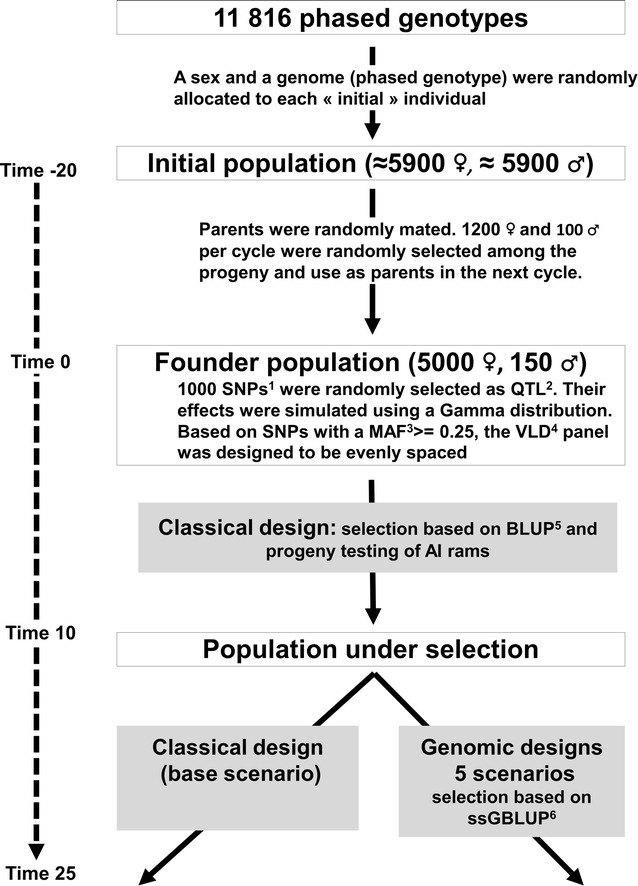
General overview of the simulation steps. ^1^SNP, single nucleotide polymorphism; ^2^QTL: quantitative trait locus; ^3^MAF: minor allele frequency; ^4^VLD: very low density; ^5^BLUP: best linear unbiased prediction; ^6^ssGBLUP: single step genomic BLUP

To compare different designs of breeding programs, we developed a stochastic simulation model where individuals, including their genome, were simulated. Stochastic events were simulated using the NAG FORTRAN library [the Numerical Algorithms Group (NAG), Oxford, UK]. Parents were randomly mated and replacements randomly selected during 20 reproductive cycles to establish a founder population. Ten years of selection for a maternal trait were then simulated by applying a classical progeny test design. At this time (Time = 10), the average LD (r^2^) between SNPs was equal to 0.13 at 50 kb, 0.07 at 200 kb and 0.05 at 1000 kb. These values of r^2^ were within the range of estimated r^2^ and Ne reported by Kijas et al. [36] for the Merino breed (r^2^ ~ 0.10 at 50 kb, Ne = 900), the Suffolk breed (r^2^ ~ 0.13, Ne = 569) and the Poll Dorset breed (r^2^ ~ 0.19 at 50 kb, Ne = 318). The next 15 years were simulated by applying either a classical or a genomic design. The main steps of the simulation are described in Fig. 1.

### Founder population

A total of 11,816 real genotypes of 54,241 bi-allelic SNPs (Illumina OvineSNP50 BeadChip-Illumina ©) from progeny-tested sires of the dairy Lacaune sheep breed were used to initialize population genomes (initial population). The genotypes were cleaned (call frequency ≥ 0.95, Hardy–Weinberg equilibrium, Mendelian inheritance compatibilities) and only autosomal SNPs with a known position on the genome were retained: 47,706 SNPs were used for the genome simulations. Using genealogical information, phased chromosomes were obtained from Fimpute [37]. At Time -20, the phased genome of the 11,816 individuals and a randomly assigned sex were used as a starting point for each simulation run.

Accuracy of genomic predictions depends on the relationships between individuals of the reference population and candidate animals [31, 38]. To obtain the genomes of a small population of females mated to a limited number of males, random mating cycles were performed using numbers of males and females close to the replacement rates used in selection in the subsequent cycles. The number of random mating cycles has a limited effect on LD (differences in LD between various numbers of cycles were lower than 0.01 at 50 kb, 200 kb and 1000 kb within 10 to 30 cycles). To obtain the desired demographic structure, 20 random mating cycles were chosen. At each cycle, 1200 females were randomly selected from females that were born in the previous two cycles and randomly mated to 100 males that were selected among males born in the previous cycle. Given the number of progeny per dam allocated according to a prior distribution (single = 0.4, twin = 0.5, triplet = 0.1), selection from two cycles was required to generate a sufficient number of female replacements. The parental gametes were generated to produce new individual genomes: the number of crossovers was simulated following a Poisson distribution $P\left( \lambda \right)$ with *λ* the chromosome length expressed in Morgan. Positions of the crossovers were allocated following a uniform distribution along the chromosome and a parental strand was randomly chosen (i.e. paternal or maternal) to start the haplotype reconstruction of the gamete. Mutations were simulated with a mutation rate of 1 × 10^−6^ per SNP per meiosis. At Time 0, founders were randomly selected according to a demographic structure (i.e. percentage of individuals per age-category) among individuals that were created in the last seven cycles, aged from 1 to 7 years old at Time 0.

### Quantitative trait loci and phenotype simulation

One thousand quantitative trait loci (QTL) underlying the maternal trait under selection were randomly selected among SNPs with a minor allele frequency higher or equal to 0.05. Following Hayes and Goddard [39], QTL effects were drawn from a Gamma distribution with a shape parameter of 0.4 and a scale parameter of 1.66. Assuming (1) no dominance effect, (2) Hardy–Weinberg equilibrium and (3) all the additive variance is explained by the QTL, the additive genetic variance was *VA* = ∑ _*i*=1_^*nqtl*^2*p*_*i*_(1 − *p*_*i*_)*q*_*i*_^2^ [40], where *p*_*i*_ denotes the allele frequency of the first allele for QTL *i* and *q*_*i*_ its effect. The QTL effects were rescaled to make the additive variance *VA* equal to 1. The maternal permanent effects, *pe*, were drawn from a normal distribution with mean 0 and variance $\frac{{rep - h^{2} }}{{h^{2} }}$ where *rep* = 0.5 and *h* ^2^ = 0.25 denote the repeatability and the heritability of the trait. The residual effects were drawn from a normal distribution with mean 0 and variance $\frac{1 - rep}{{h^{2} }}$. The *k*th phenotype *y*_*jk*_ of a female *j* was simulated as *y*_*jk*_ = *TBV*_*j*_ + *pe*_*j*_ + *e*_*jk*_ with *TBV*_*j*_ the true breeding value depending on the effects of all QTL and the female genotype.

### Estimation of breeding values

The estimation of breeding values was based on an animal best linear unbiased prediction (BLUP) model [41] for the classical design and genomic evaluation on an animal single-step genomic (G)BLUP [42] for the genomic design. Both types of evaluation were performed using the Blupf90 software [43] developed by Misztal et al. The genomic relationship matrix was built following Van Raden [44]. Marker inconsistencies between parents and progeny were due to imputation errors. Errors detected by Blupf90 resulted in the removal of the corresponding progeny genotype before the evaluation. Coefficients of inbreeding based on pedigree information were computed using the methodology developed by Aguilar and Misztal [45] and implemented in the Blupf90 software.

### Very low-density and medium-density panels

SNPs with a minor allele frequency higher or equal to 0.25 and not selected as a QTL were considered as candidates for inclusion in the VLD panel. To select *n*_*vld*_ evenly spaced SNPs (*n*_*vld*_ = 250, 500 and 1000), the genome was divided into *n*_*vld*_ windows. Each window was subdivided into three parts and a SNP was randomly selected from candidate SNPs located in the central part. If no candidate was available, a SNP located within the window or in adjacent windows was selected. The MD panel (46,706 SNPs) included all SNPs except those selected as QTL.

### Population and breeding designs

**Fig. 2:**
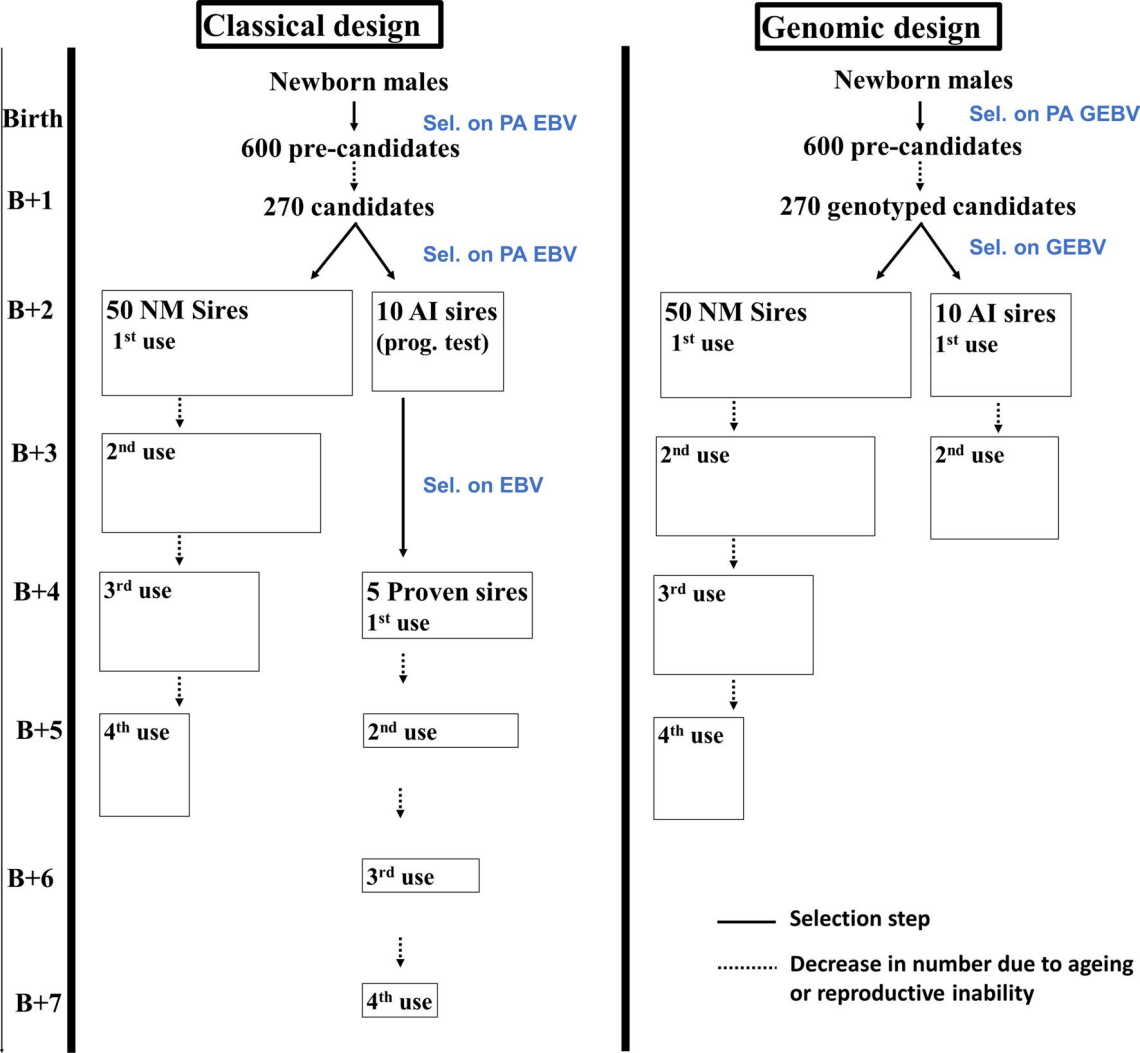
General overview of the classical and genomic designs. Sel. on PA EBV: truncation selection on parent average estimated breeding values; Sel. on EBV: truncation selection on estimated breeding values; Sel. on PA GEBV: truncation selection on parent average genomic estimated breeding values; Sel. on GEBV: truncation selection on genomic estimated breeding values; prog. test: males in progeny testing using artificial insemination (AI); Proven sires: AI sires selected on progeny testing; NM sires: natural mating sires; AI sires: artificial insemination sires

Around 5000 females divided into 10 flocks were recorded each year for the trait under selection. According to Hill [46], and neglecting the correlation between progeny sizes of sires to breed sires and sires to breed dams and between progeny sizes of dams to breed sires and dams to breed dams, the effective population size was around 180. At each reproductive cycle (length = 1 year), half of the breeding females were selected on EBV for mating by AI. Assuming a fertility rate of 55% on induced estrus, females that did not conceive to AI and females not selected for AI were then randomly mated to natural mating sires allocated to their flock assuming a fertility rate of 90%. The average number of females per naturally-mated sire was equal to 35. To prevent inbreeding, a male (AI or natural mating) could not be mated to a female belonging to its dam’s flock. The number of progeny per dam depended on the reproduction mode (induced or natural estrus) and parity (first versus second and more). At each cycle, some dams were randomly culled with a proportion varying from 0.10 after parity 1 to 0.50 on average after parity 6. The maximum parity was 7 and around 24% of dams were replaced per cycle. Replacement females, allocated to their dam’s flock, were first randomly chosen among females that were born from an AI sire and then among females that were born from a natural mating sire. There is no difference between the classical and genomic designs, presented in Fig. 2, regarding how females were selected.

About 600 male candidates were preselected among newborn males (~ 3500) based on the parental mean EBV in the classical design and on the parental mean GEBV in the genomic design. In practice, all candidates were born from proven AI sires (classical design) and genomic AI sires (genomic design) given their genetic superiority. About 10% of male candidates died within their first year. At 1 year of age, 50% of the male candidates were culled for non-genetic factors. In the classical design, the ten candidates with the highest parental mean EBV (out of 270 candidates) were selected and mated across flocks by AI to 1100 females (110 per male on average) for progeny-testing. Two cycles later, the five AI sires with the highest EBV (including progeny records) were selected as proven AI sires and used at most for four cycles (from 4 to 7 years old). At each cycle, 1400 females were mated to proven AI sires. In the genomic design, MD or imputed MD genotypes of candidates were available. The ten candidates with the highest GEBV were selected as AI males and mated across flocks to 1100 females. Genomic AI sires were used at most for another cycle and mated to 1400 females. To mimic the variation in the AI dose production per male, the inseminated dams were randomly allocated to one AI sire with a maximum number of AI per male equal to 140 for 2-year old sires and to 250 for older sires. About 50 naturally-mated male replacements were selected among 260 candidates not selected as AI sires, based on their parental mean EBV in the classical design or on their GEBV in the genomic design. These naturally-mated sires were used at most for four cycles (from 2 to 5 years old).

Five genomic scenarios (Table 1) were assessed. Some scenarios included an imputation step to reconstruct the MD genotypes of individuals genotyped with the VLD panel. The imputation was performed using the software Fimpute developed by Sargolzaei et al. [37]. The reference population for imputation was based first on the historical population of AI and natural mating sires at Time 10: about 50 progeny tested sires (each with ~ 30 daughters), 50 proven sires (~ 125 daughters) and 400 sires (~ 9 daughters). From Time 10 to 25, new selected sires were added, i.e. about 10 AI and 50 natural mating sires per year. The scenarios differed in the choice of genotyped animals (including dams or not), the SNP panel used for the genotyping of male candidates and dams (MD or VLD followed by imputation to MD) and the information used for imputation (including pedigree or not). In the GSsc scenario, sires and male candidates are genotyped using the MD panel. In the GSs_Ic scenario, sires are genotyped using the MD panel and male candidates using the VLD panel. In the GSscd scenario, sires, male candidates and dams are genotyped using the MD panel. In the GSs_Icd scenario, sires are genotyped using the MD panel and male candidates and dams using the VLD panel. To assess the importance of pedigree information in the genomic design, the last scenario, GSs_Icd_pop, was similar to GSs_Icd but an imputation method that ignored pedigree information (population based) was used.

**Table 1:** Information taken into account for (genomic) breeding value estimation and imputation step according to scenarios

Scenarios^a^	CS^b^	GSsc^c^	GSs_Ic^c^	GSscd^c^	GSs_Icd^c^	GSs_Icd_pop^c^
Genotypes (G^d^) or imputed genotype (IG^e^)						
Sires		G	G	G	G	G
Male candidates		G	IG	G	IG	IG
Dams				G	IG	IG
Imputation methodology: population (P)/familial (F)			P + F		P + F	P

^a^Phenotypes and pedigree were included in all scenarios

^b^CS = classical selection design

^c^GS = genomic selection design; GSsc, sires and candidates had medium-density genotypes; GSs_Ic, sires had medium-density genotypes and candidates had medium-density genotypes imputed from very low-density genotypes; GSscd, sires, candidates and dams had medium-density genotypes; GSs_Icd, sires had medium-density genotypes and candidates and dams had medium-density genotypes imputed from very low-density genotypes; GSs_Icd_pop, sires had medium-density genotypes and candidates and dams had medium-density genotypes imputed from very low-density genotypes without using the pedigree information

^d^Medium-density genotypes (46 K)

^e^Medium-density genotypes (46 K) imputed from very low-density genotypes (≤ 1000)

### Simulation outputs

At each cycle and for each scenario, the average TBV and inbreeding coefficient of females in their first parity were computed. The annual genetic gain and annual rate of inbreeding were estimated as the regression slopes of the average TBV and average inbreeding coefficient of first parity females on time between cycles 10 and 25. The means and standard deviations presented are based on 50 replicates. At time 25, we computed the average Pearson correlation between the TBV and the (G)EBV for dams and male candidates as well as the average concordance rate of imputed genotypes (the number of correctly imputed alleles divided by the number of imputed alleles) of dam and male imputed genotypes.

## Results

Table 2 includes the annual genetic gain, the annual increase in inbreeding, (G)EBV accuracies, and concordance rates of imputed dams and male candidates for six designs. Computed at time 25, we assumed that GEBV accuracies and concordance rates were close to the upper bounds of values obtained over the simulation time. We used a VLD panel of 1000 SNPs for designs that included an imputation step to the MD panel. Table 3 provides the same information for designs based on female imputed genotypes using panels of 1000, 500 or 250 SNPs.

**Table 2:** Genetic gain, inbreeding rate, (G)EBV accuracies, and imputation concordance rates for six scenarios^a^ with VLD panels of 1000 SNPs (standard deviations for 50 replicates shown in brackets)

Scenarios^a^	CS^b^	GSsc^c^	GSs_Ic^c^	GSscd^c^	GSs_Icd^c^	GSs_Icd_pop^c^
Genetic gain^d^ (σ_a_/year)	0.162 (0.015)	0.205 (0.019)	0.197 (0.021)	0.249 (0.016)	0.230 (0.014)	0.179 (0.020)
Inbreeding rate/year^e^	0.0043 (0.0011)	0.0034 (0.0006)	0.0033 (0.0006)	0.0028 (0.0005)	0.0031 (0.0006)	0.0040 (0.0008)
(G)EBV accuracy^f^						
Dams^g^	0.71 (0.02)	0.76 (0.02)	0.75 (0.02)	0.87 (0.01)	0.83 (0.02)	0.74 (0.03)
Male candidates	0.36 (0.07)	0.53 (0.06)	0.51 (0.06)	0.71 (0.05)	0.63 (0.05)	0.43 (0.07)
Imputation concordance rate^h^						
Dams^g^					96.1 (0.1)	91.1 (0.2)
Old females^i^					93.3 (0.1)	88.3 (0.2)
Male candidates			94.7 (0.2)		96.5 (0.1)	92.3 (0.2)

^a^Scenarios based on imputation were performed with a very low-density 1000-SNP panel

^b^CS = classical selection design

^c^GS = genomic selection design; GSsc, sires and candidates had medium-density genotypes; GSs_Ic, sires had medium-density genotypes and candidates had medium-density genotypes imputed from very low-density genotypes; GSscd, sires, candidates and dams had medium-density genotypes; GSs_Icd, sires had medium-density genotypes and candidates and dams had medium-density genotypes imputed from very low-density genotypes; GSs_Icd_pop, sires had medium-density genotypes and candidates and dams had medium-density genotypes imputed from very low-density genotypes without using the pedigree information

^d^Computed as the slope of the average true breeding value of females in first parity between time10 and time25

^e^Computed as the slope of the average inbreeding coefficient of females in first parity between time10 and time25

^f^Computed as the average Pearson correlation between the true breeding value and (genomic) estimated breeding values of animals at time25

^g^Dams mated at time 25

^h^Computed as the average of number of correctly imputed SNP divided by the number of imputed SNP obtained for the imputation realized at time 25

^i^Dams not present anymore at time 25

**Table 3:** Genetic gain, inbreeding increase, (G)EBV accuracies, and imputation concordance rates for the GSs_Icd genomic design using VLD densities of 250, 500, and 1000 SNPs (standard deviations for 50 replicates shown in brackets)

Scenarios	GSs Icd^a^	GSs Icd^a^	GSs_Icd^a^
Number of SNPs	1000	500	250
Genetic gain^b^ (σ_a_/year)	0.230 (0.014)	0.183 (0.016)	0.175 (0.015)
Inbreeding^c^	0.0031 (0.0006)	0.0037 (0.0007)	0.0040 (0.0010)
GEBV accuracy^d^			
Dams^e^	0.83 (0.02)	0.74 (0.02)	0.73 (0.03)
Male candidates	0.63 (0.05)	0.45 (0.07)	0.38 (0.02)
Imputation concordance rate^f^			
Dams^e^	96.1 (0.1)	91.8 (0.3)	87.3 (0.4)
Old females^g^	93.3 (0.1)	88.7 (0.2)	84.5 (0.3)
Male candidates	96.5 (0.1)	92.4 (0.3)	88.0 (0.4)

^a^GSs_Icd = genomic selection design, sires had medium-density genotypes and candidates and dams had medium-density genotypes imputed from very low-density genotypes

^b^Computed as the slope of the average true breeding value of females in first parity between time10 and time25

^c^Computed as the slope of the average inbreeding coefficient of females in first parity between time10 and time25

^d^Computed as the average Pearson correlation between the true breeding value and (genomic) estimated breeding values of animals at time 25

^e^Dams mated at time 25

^f^Computed as the average of number of correctly imputed SNP divided by the number of imputed SNP obtained for the imputation realized at time 25

^g^Dams not present anymore at time 25

### Annual genetic gain

Annual genetic gains indicated in Tables 2 and 3 correspond to the slope of the average TBV of females in parity 1 from year 10 to 25 for each design. Results show that the genetic gain for a genomic design based on a sire reference population (GSsc: 0.205$\upsigma_{\text{a}}$) was 27% higher than for the classical design (CS: 0.162$\upsigma_{\text{a}}$). When male candidates were imputed from the VLD, the genetic gain slightly decreased but remained significantly different from that of the classical design (GSs_Ic: 0.197$\upsigma_{\text{a}}$). Including dams in the reference population doubled the additional genetic gain of the genomic design (+ 54%) compared to the classical design (GSscd: 0.249$\upsigma_{\text{a}}$). When candidates and dams were imputed, based on both family and population information, the increase in gain was lower (GSs_Icd: 0.230$\upsigma_{\text{a}}$) but remained higher than that of a genomic design based on a sire reference population (GSs_Ic: 0.197$\upsigma_{\text{a}}$). When imputation was based on population information, the genetic gain (GSs_Icd_pop: 0.179$\upsigma_{\text{a}}$) was close to the gain achieved in the classical design.

### Inbreeding

The rates of inbreeding shown in Tables 2 and 3 correspond to the slope of the average inbreeding coefficient of females in parity 1 from year 10 to 25 for each design. Apart from when the imputation method did not take pedigree information into account, the increase in inbreeding was significantly slower for the genomic scenarios (from 0.0028 to 0.0034 per year) than for the classical design (0.0043 per year). Within genomic scenarios, the increase in inbreeding per year was lower as the number and quality of genotypes increased.

### GEBV accuracies and imputation concordance rate

The EBV accuracies shown in Tables 2 and 3 were calculated as the average Pearson correlation between TBV and (G)EBV of females in parity 1–7 (dams) and young male candidates at time 25. Accuracies for dams were slightly higher in the genomic design based on a sire reference population (GSsc: 0.76) than in the classical design (CS: 0.71). Including dams in the reference population resulted in a substantial increase in accuracy either when they were genotyped with MD (GSscd: 0.87) or genotyped with VLD and imputed to MD using population and family information (GSs_Icd: 0.87). When pedigree information was not used for imputation, the GEBV accuracy for dams was lower (GSs_Icd_pop: 0.74) than in the genomic design without dams in the reference population. Accuracy for male candidates was lower in the classical design (CS: 0.36), since they were selected on mid-parent EBV, compared to the genomic designs where their own genomic information was included. For these males, accuracies increased from 0.43 to 0.71 as the quality of their own genomic information (imputed genotypes based either on population or population and family information, MD genotypes) increased and by including dams in the reference population.

The concordance rates of imputation reported in Tables 2 and 3 correspond to the number of correctly imputed alleles divided by the number of imputed alleles at Time 25. Results show that dams and young male candidates are imputed with higher accuracy than old females (+ 3%). Adding pedigree information (GSs_Icd) resulted in a higher imputation accuracy of + 4.2% for male candidates and + 5.0% for females compared to ignoring the pedigree in the imputation process (GSs_Icd_pop). Adding female VLD genotypes resulted in a gain of + 1.8% for male candidates.

### Effect of the number of SNPs in the VLD panel on the GSs_Icd design

Table 3 shows that the genetic gain was much affected by the number of VLD SNPs used for imputation. The additional gain obtained for 1000 SNPs (0.230$\upsigma_{\text{a}}$) fell to 0.183σ_a_ for 500 SNPs, while the gain for 250 SNPs was close to, but still significantly higher (0.175$\upsigma_{\text{a}}$) than, the genetic gain obtained for the classical design (0.162$\upsigma_{\text{a}}$). Compared to the use of 1000 SNPs, the decrease in accuracy was approximately 0.1 for dams irrespective of the number of SNPs, 500 or 250. These accuracies were close to the accuracy obtained when dams were not included in the reference population (GSsc, GSs_Ic). The same comparison for male candidates shows that the decrease was larger when 250 SNPs (− 0.25) were used than when 500 SNPs (− 0.18) were used. Across all numbers of SNPs, the magnitude of the decrease in concordance rate was similar regardless of the category considered: from − 4.1 to − 4.6 for 500 SNPs and from − 8.5 to − 8.8 for 250 SNPs compared to 1000 SNPs.

## Discussion

In this study, our reference scenario the classical design produced a rate of genetic gain of 0.162$\upsigma_{\text{a}}$. This result is close to 0.173$\upsigma_{\text{a}}$, which is the annual genetic gain from 1986 to 1999 estimated from real data for the Red Faced Manech breed [47], a breeding program similar to the simulation design (single trait selection until 2003). Compared to the classical design, genomic designs generated more annual genetic gain and limited the increase in inbreeding. Sensitivity to a lower heritability (h^2^ = 0.10 instead of 0.25) and a higher efficiency of the classical design (proportion selected of 0.33 instead of 0.5 to select proven AI sires) were also assessed. Based on ten replicates (results not shown), differences in genetic gain were slightly smaller for a low heritability or a higher efficiency of the classical design, but were of a similar magnitude to the results obtained with the classical design, GSscd and GSs_Icd in this study.

The rate of increase in inbreeding was lower in genomic scenarios for three main reasons: first, the average contribution of natural mating sires was identical across designs whereas the average contribution of AI sires was smaller in the genomic design (genomic AI sires were systematically culled after they had been used for two cycles); second, the selection intensity applied on parent average genetic value to select male candidates in the genomic design was lower than that to select AI and natural mating sires in the classical design; and third, genomic information was expected to reduce the relative importance of pedigree information in breeding value estimation through improved estimation of the Mendelian sampling term [17]. The latter might explain why within genomic scenarios, the increase in the rate of inbreeding was lower as the quality and quantity of the genomic information improved.

### Performance of a genomic selection design based on a reference population of related sires

Compared to the classical design, a genomic design based on a reference population of related sires resulted in an increase in genetic gain of 26.3% per year when candidates were genotyped with the MD panel (GSsc). This increase is inferior to the increase in gain of 50% calculated by Shumbusho et al. [1] for the Red Faced Manech breed using a deterministic model. Although the designs were similar, Shumbusho et al. [1] computed the accuracy of GEBV based on a reference population of 2000 animals with a constant number of records using the formula of Daetwyler et al. [48], whereas in our study, the cumulated number of genotyped sires (included in the single-step GBLUP) increased from around 500 (Time 10) to 1250 (Time 25), and the number of daughters per sire was highly variable due to both use of AI and natural mating. For the GSsc scenario, the increase in accuracy of GEBV was moderate for dams (7%) but considerable for young males (47%). When candidate male genotypes were imputed from genotypes based on 1000 SNPs (GSs_Ic), the increase in gain (+ 21.6%) compared to that obtained with the classical design was lower than with GSsc. The average concordance rate, equal to 94.7%, is of the same order as results obtained by Wang et al. [25] and corroborates that an acceptable imputation quality based on a VLD panel could be achieved provided that sires are included in the training population. The GEBV accuracies for male candidates were slightly lower for GSs_Ic than for GSsc (*P* = 0.068) in agreement with results obtained in sheep by Moghaddar et al. [31] for a similar range of imputation accuracies.

Sires of male candidates were selected among young males in the genomic design and progeny-tested males in the classical design. Although the accuracy of GEBV of young males was lower than that of progeny-tested males (0.71—results not shown), higher selection intensities and a shorter average generation interval resulted in a higher genetic gain. Regardless of the design, male candidates were preselected based on parent average genetic values. The genomic AI sires were selected on their own genomic value before 2 years of age with a proportion selected of 1/27 versus 1/2 at 4 years old after progeny-testing for AI sires in the classical design. Since genomic AI sires were used for a maximum of 2 years, the average generation interval was reduced (3 years instead of 4). The natural sires were used and selected at the same age with a proportion selected close to 1/5 but on their own genomic value in the genomic design or on parent average genetic value in the classical design.

### Value of female VLD genotypes

The comparison between scenarios with (GSscd) or without females (GSsc) included in the reference population shows that adding dam genotypes, along with their phenotypes, resulted in a doubling of the increase in genetic gain of the genomic design. Genotyping dams led to an increase in the average accuracy of GEBV for both dams (14%) and male candidates (34%). The effect of including cow genotypes in the reference population on accuracy and bias of genomic prediction has been widely reported in dairy cattle [11–15, 49–55]. Genetic gain varied depending on the population and reference population structure. For example, Koivula [14] reported a small additional gain (increase in accuracy of + 2 to 4%) when the reference population contained about 4400 sires, whereas McNugh [11] reported that including a large female population in the reference population increased the annual genetic gain by a factor of 3. In our study, the increase in gain was also large. Buch et al. [12] and Gonzalez-Recio et al. [13] showed that the increase in genomic prediction accuracy due to female information was most important when phenotypes and sizes of sire progeny groups were limited. Thus, this result was expected given the structure of our reference population: the number of sires was limited and most sire progeny groups were generated by natural mating, resulting in small numbers of daughters and thus inaccurate phenotypes.

When male candidates and dams were genotyped with a 1000-SNP panel and imputed genotypes were included for genomic evaluation (GSs_Icd), the additional genetic gain was reduced but it remained higher (+ 41%) than that obtained with the classical design. With a concordance rate of imputed animals of about 96%, the accuracy of GEBV of male candidates and dams decreased compared to that in GSscd (respectively − 11.3 and − 4.6%) but remained higher than accuracies obtained with a reference population based on sires (respectively + 15.9% and + 8.4% pts). Compared to the classical design, the increase in accuracy with a 1000-SNP panel was + 41% but this decreased to + 8% with a 250-SNP panel. Lower imputation accuracies obtained with lower density SNP panels and larger numbers of genotypes discarded from the genomic evaluation due to parent-progeny mismatches (results not shown), resulted in no increase in GEBV accuracy of dams when they were genotyped with the 250- or 500-SNP panel: both panels gave the same accuracies as the GSsc scenario in which dams were not included in the reference population. For male candidates, the accuracy of GEBV was lower for 500 (− 40.0%) and 250 (− 65.8%) SNPs than the accuracy obtained with 1000 SNPs but the increases in gain for genomic scenarios based on 250 and 500 SNPs, compared to the classical design, were highly significant (*P* < 0.001). Comparing GSs_Icd based on 250 SNPs with the classical design, the differences in accuracy of GEBV for dams and male candidates were small (+ 0.02) but highly significant (*P* < 0.001) for dams and moderate for male candidates (*P* = 0.0565).

### Importance of using pedigree information in the imputation of MD genotypes from VLD genotypes

In GSs_Icd_pop, we removed pedigree information in the imputation of male candidate and dam genotypes. The comparison of GSs_Icd_pop with GSs_Icd shows that removing pedigree information in the imputation step decreased the concordance rate by 5 points and 4.2 points, respectively for dams and male candidates. A high proportion of genotypes (around 80%) were removed in GSs_Icd_pop, due to mismatches between parents and progeny (results not shown). In GSs_Icd_pop, the annual genetic gain remained higher (+ 9.5%) than in the classical design but was substantially lower than in GSs_Icd (+ 45%). The contribution to the analysis of co-segregation between VLD and MD SNPs has already been reported in previous studies which highlight the beneficial effect of including relatives in the reference population for imputation [21, 24, 26, 27, 29–31, 34], especially sires and grandsires [24]. The effect of including close relatives in the reference population on genomic prediction accuracy was also pointed out by previous studies on simulated and real data [38, 56]. Habier et al. [57] showed that co-segregation, as well as LD and additive genetic relationships, all contribute to the capture of QTL effects, whereas Sun et al. [58] showed that explicitly modelling the co-segregation results in higher accuracy of genomic prediction when the recent effective population is small. Such results suggest that a small reference population that contains all dams and sires of candidates can result in substantial additional genetic gain based on a better genomic prediction accuracy.

### Feasibility of a genomic design including imputed genotypes on dams in the reference population

Higher gain was obtained with a genomic design when including dam medium-density genotypes in the reference population. The economic value of such a scenario should be assessed over the whole population, including both the nucleus and commercial levels. However, as discussed for Australian sheep and beef cattle industries by van der Werf and Banks [16], for an individual breeder or nucleus, the main objective is to achieve the maximum economic return within the company. Regarding the value of breeding stock, a lower genetic gain but cheaper design can be relevant for sheep breeding programs. For a French small ruminant breeding program, the cost of AI males including their progeny testing is assumed to be at least 400 euros per male per year [59]. In a genomic selection design, due to AI sires being used earlier and over a shorter period, the corresponding cost would be reduced, but genotyping costs have to be considered for building the reference population and identifying candidates. Implementing a GSscd design would require a major investment compared to the current classical design since all dams have to be genotyped. The investment for implementing a GSs_Icd design would be the same as that for the classical design, for example if VLD genotypes of dams were supported by breeders for parentage assignment purposes. However, the current price of the very low-density panel available for sheep (around 20 euros) limits its use for parentage assignment by breeders. A combined use of a VLD panel for both parentage and genomic selection might be cost-effective.

Plieschke et al. [15] noted that including female genotypes led to increased accuracy of GEBV without bias as long as the females chosen for genotyping were randomly sampled. In our study, all dams were genotyped. The main purpose was to assess the usefulness for genomic selection of VLD genotypes used for parentage assignment. From a practical point of view, one can imagine that only a certain proportion of flocks will use parentage assignment based on SNPs. In that case, there could be variation across flocks, and potentially within a flock, of the average dam GEBV accuracy according to the VLD genotyping status of dams. This could result in a bias since male candidates are selected on parent mean GEBV.

## Conclusions

Using a stochastic model, we compared classical and genomic selection designs. Five genomic scenarios were assessed by varying the structure of the reference population (with or without genotyping females) and the genotyping panels that were used (medium density, or imputed to medium from very low density). Compared to the classical design with progeny testing, genomic scenarios generated more genetic gain and limited the increase in inbreeding. This superiority was based on higher selection differentials that are applied to male candidates to select sires for both AI and natural mating. The combined use of very low-density genotypes for male candidates and dams together with imputation resulted in lower genetic gain than scenarios based on medium-density genotypes. However, the increase in gain was substantial compared to that in the classical design. Using very low-density SNP panels might be more profitable at the nucleus level given its lower cost relative to medium- or low-density SNP panels and multipurpose use (parentage assignment and genomic selection).
